# Bacterial ligands as flexible and sensitive detectors in rapid tests for antibodies to SARS-CoV-2

**DOI:** 10.1007/s00216-022-03939-2

**Published:** 2022-02-11

**Authors:** Simone Cavalera, Fabio Di Nardo, Matteo Chiarello, Thea Serra, Barbara Colitti, Cristina Guiotto, Franca Fagioli, Celeste Cagnazzo, Marco Denina, Annagloria Palazzo, Fiora Artusio, Roberto Pisano, Sergio Rosati, Claudio Baggiani, Laura Anfossi

**Affiliations:** 1 Department of Chemistry, University of Turin, via Pietro Giuria 5, Turin, TO Italy; 2 Department of Veterinary Sciences, University of Turin, Turin, TO Italy; 3 A.O. Ordine Mauriziano, Ospedale Umberto I di Torino, Turin, TO Italy; 4 Department of Public Health and Paediatrics, Regina Margherita Children’s Hospital, University of Turin, Turin, TO Italy; 5 Department of Public Health and Paediatrics, Infectious Diseases Unit, Regina Margherita Children’s Hospital, Turin, TO Italy; 6 Department of Medical Sciences, Molinette General Hospital, Infectious Diseases Unit, Turin, TO Italy; 7 Department of Applied Science and Technology, Politecnico di Torino, Turin, TO Italy

**Keywords:** Broad-specific ligands, Serological testing, Design of experiment, Gold nanoparticles, Lateral flow immunoassay

## Abstract

Lateral flow immunoassay (LFIA) is widely employed as point-of-care tests (POCT) for the diagnosis of infectious diseases. The accuracy of LFIA largely depends on the quality of the immunoreagents used. Typical LFIAs to reveal the immune response to severe acute respiratory syndrome coronavirus-2 (SARS-CoV-2) employ anti-human immunoglobulin (hIG) antibodies and recombinant viral antigens, which usually are unstable and poorly soluble. Broad selective bacterial proteins, such as Staphylococcal protein A (SpA) and Streptococcal protein G (SpG) can be considered alternatives to anti-hIG to increase versatility and sensitivity of serological LFIAs because of their high binding capacity, interspecies reactivity, and robustness. We developed two colorimetric LFA devices including SpA and SpG linked to gold nanoparticles (GNP) as detectors and explored the use of a specific, stable, and soluble immunodominant fraction of the nucleocapsid protein from SARS-CoV-2 as the capturing agent. The optimal amount of SpA-GNP and SpG-GNP conjugates and the protein-to-GNP ratios were defined through a full factorial experimental design to maximize the diagnostic sensitivity of the LFIAs. The new LFA devices were applied to analyze 105 human serum samples (69 positive and 36 negatives according to reference molecular diagnostic methods). The results showed higher sensitivity (89.9%, 95% CI 82.7–97.0) and selectivity (91.7%, 82.6–100) for the SpA-based compared to the SpG-based LFA. In addition, 18 serum samples from cats and dogs living with COVID-19 patients were analyzed and 14 showed detectable levels of anti-SARS-CoV-2 antibodies, thus illustrating the flexibility of the SpA- and SpG-based LFAs.

**Graphical abstract:**

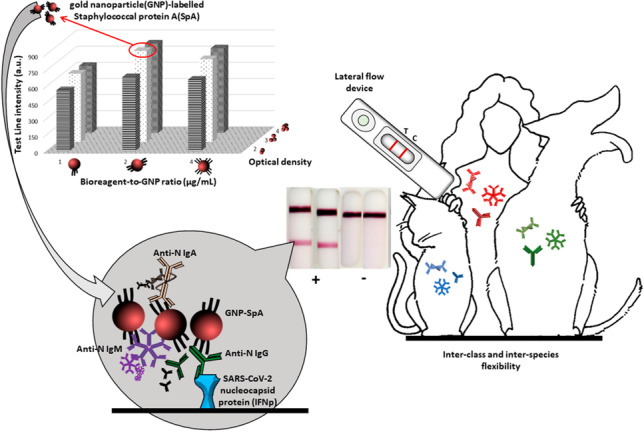

**Supplementary Information:**

The online version contains supplementary material available at 10.1007/s00216-022-03939-2.

## Introduction

After 2 years from the outbreak of the novel coronavirus, SARS-CoV-2 pandemic, the situation is still on the razor-edge. Socially and economically exhausted countries are still oscillating between lockdowns and re-openings of commercial activities. More than 5.5 million people died, and almost 350 million infected people have been confirmed worldwide [1]. Early detection of SARS-CoV-2 is one of the crucial interventions to control virus spread and dissemination [2]. Among the possible diagnoses of SARS-CoV-2, targeting diverse biomarkers (e.g. viral RNA, spike protein, nucleocapsid protein, anti-SARS-CoV-2 antibodies), the direct methods such as molecular assays and antigen assays have been the gold standard to directly detect for the presence of SARS-CoV-2. Nevertheless, the importance of serology in monitoring the immune response to SARS-CoV-2 was demonstrated and underlined [3, 4]. Typical laboratory-based serological analyses are performed by enzyme-linked immunosorbent assay, immunofluorescent assay, and chemiluminescence immunometric methods [5, 6]. The analytical performances achieved by serological laboratory tests are, generally, performant in terms of sensitivity and specificity [7]. Many serological rapid tests have been also validated and put on the market, exploiting the lateral flow immunoassay (LFIA) format. Considering the importance of the screening and the usefulness of the serosurvey, high-performance LFIAs have been developed and commercialized in the latest months. It is worth noting that the trend to discriminate between anti-SARS-CoV-2 immunoglobulin M (IgM) and immunoglobulin G (IgG), typical of the spring 2020 rapid serological test generation, has been largely explored but it is still under discussion. In particular, the role of the IgM is unclear, and the scientific opinion is split between those considering them as predictive of early infection [8] and those stating the opposite [9–11]. The detection of the total antibody response to a SARS-CoV-2 is an interesting new trend, including also other classes of antibodies besides IgG and IgM, such as immunoglobulins A (IgA) [12]. In a previous work, we demonstrated the high sensitivity and specificity reached by using the total antibody approach [13]. In other studies, the effects of using Staphylococcal protein A (SpA) and Streptococcal protein G (SpG) as detection [14, 15] or capturing elements [16] on antibody testing have been explored. Another interesting benefit of bacterial bioligands is their broad selectivity towards immunoglobulins of different animal species. The possibility to detect anti-SARS-CoV-2 antibodies in pets can help in understanding the role of companion animals as hosts and reservoirs for the infection. The use of SpG and SpA as detection ligands provides the required flexibility to apply the same LFA device to detect the serological response in different animal species. Generally, such bioligands are used for purification of immunoglobulins [17]. SpA is a broad-specific reagent, able to bind to several classes of immunoglobulins, with high affinity to IgG but also cross-reacting with IgA, IgM, and immunoglobulins D. SpG specifically binds to the IgG [18] class but with a broad interspecies cross-reactivity. SpA can bind to up to five immunoglobulins at the same time [19], showing high capacity, while SpG shows higher affinity to IgG on respect to SpA. Both show high affinity to the Fc fragment. However, they bind also to the Fab region (SpA generally very weakly [20], SpG moderately). The use of SpA and SpG as probes in LFA has been reported in several scientific works and for commercially available devices [15, 16, 21].

In this work, we propose two LFA devices able to detect the immune response to SARS-CoV-2 in humans and pets by using SpA and SpG labelled with gold nanoparticles (GNPs) as the probes.

This approach does not involve any specific antibodies as bioreagents for capture or detection, making the LFA generally applicable. The use of bacterial bioligands, in fact, trespasses the limitations of the typical antibody LFAs, in terms of the class of antibodies detected and of the animal species producing those antibodies.

To this aim, we investigated preliminarily the effect of varying the bioligand-to-GNP ratio and the amount of the probe, intended as the quantity of bioligand-GNP probe measured by the optical density (OD) of GNPs. We considered the maximal signal intensity and the absence of background signals as criteria to judge results. In addition, the capturing reagent applied to form the test line was a recombinant C′-terminal subunit of the nucleocapsid protein (IFNp), previously identified as a specific and immunodominant fraction of SARS-CoV-2 protein (personal observation, unpublished). The IFNp was selected since it did not overlap with nucleocapsid proteins of other coronaviruses (SARS CoV and MERS) so it was assumed as selective to SARS-CoV-2 and reasonably more convenient for future applications. On the other hand, the use of a small region rather than the whole antigen may limit the sensitivity. Therefore, we investigated the performance of the new capturing antigen and of the LFA devices including the SpA or the SpG probes (A-LFA and G-LFA, respectively). For comparison, we tested the same panel of serum samples previously used for the assessment of the device including the whole recombinant nucleocapsid protein N (N-LFA) and SpA. In that case, however, the antigen was labelled with GNP and served as detector, while SpA was used as capturing ligand [13]. The A-LFA and G-LFA devices were also applied to test 18 pet serum samples (4 belonging to dogs and 14 to cats) living in contact with SARS-CoV-2 positive owners. The concern of pet serological SARS-CoV-2 testing is still under study. Nevertheless, the risk of transmission from humans to animals, especially in close living condition, exists and has been verified in some studies and resumed by the world organization for animal health (OIE) [22–24]. According to these reports, the symptomatology affecting animal species is highly variable and this makes it more difficult to diagnose SARS-CoV-2 in the absence of a molecular nasopharyngeal swab test by real-time reverse transcriptase polymerase chain reaction (rRT-PCR). The use of broad-specific bioreagent such as SpG and SpA to set rapid screening tests allows for the expansion of their usage to detect SARS-CoV-2 in animals that, otherwise, are often left behind on respect to humans in the deal with the pandemic [25]. The main contribution of this work, on respect to the state of the art, is focused on (i) the use of the design of an experimental approach as an effective strategy to obtain efficient gold conjugates to be employed as probes in the LFIA development, (ii) the exploration of the effect achieved by changing the role of the immunoreagents (detection and capture) in sandwich-type lateral flow immunoassay, also in connection to the very different contact time between the two reagents and the sample (or the analyte), which dramatically impacts the rate of reagent-analyte complex formation and, consequently, on the overall sensitivity of the assay; (iii) the introduction of broad-specific detection bioligands as sensitive and flexible detection bioligands that can be used as general systems for developing serological LFIA and which enable the use of a single device for detecting antibodies produced in different animal species.

## Materials and methods

### Chemicals

Gold (III) chloride trihydrate (ACS reagent), Staphylococcal protein A (SpA), Streptococcal protein G (SpG), boric acid, sodium tetraborate decahydrate, tris(hydroxymethyl)aminomethane, glycine, sucrose, and bovine serum albumin (BSA) were obtained from Sigma-Aldrich (St. Louis, MO, USA). Tween 20 and other chemicals were purchased from VWR International (Milan, Italy). Nitrocellulose membranes (CNPC-SS12) with cellulose adsorbent pad and FR-1 sample pads were purchased by MDI membrane technologies (Ambala, India). Glass fibre conjugate pads were obtained from Merck Millipore (Billerica, MA, USA). Statistical calculations were carried out with SigmaPlot 11.0 software**.**

Recombinant subunit of SARS-CoV-2 nucleoprotein, corresponding to amino acid sequence 230–408, was expressed in prokaryotic vector in frame with glutathione S-transferase and affinity purified using standard techniques.

### Synthesis of the SpA_GNP and SpG_GNP conjugates

The synthesis of 40-nm-diameter GNPs at optical density 1 was carried following the citrate reduction method by Turkevich [26] as described in the SI. The synthesis of the gold conjugates was made by passive adsorption of the protein (SpA or SpG) on the surface of the citrate-capped GNPs. The flocculation test was modified starting from previous works [27]. Briefly, a salt-induced aggregation test was carried on the GNP solution after the adjustment of the pH at 6.0 with carbonate buffer (0.05 M, pH 9.6). Then, 250 μL was inserted in wells of a microtiter plate and incubated for 30 min with increasing volumes (0–25 μL) of the bioligand from 0.1 mg/mL solution in phosphate buffer (0.02 M, pH 7.4). Then, 25 μL of aqueous NaCl (10% w/v) was added and reacted for 10 min to promote aggregation of unstable GNPs. The absorbance was read at 540 nm and 620 nm by a microplate reader (Multiskan FC, Microplate Photometer). The 540 nm absorbance was related to the non-aggregated fraction of GNPs, while the absorbance at 620 nm was proportional to aggregation, as the shift of the LSPR band towards higher wavelengths is due to aggregation. The results are reported in the SI (Figure S1). The conjugation procedure is reported in the SI (Figure S2). The SpA was adsorbed using different bioreagent-to-GNP ratios. In detail, to 1 mL of GNP solution of optical density 1, 1, 2, and 4 µg of SpA and 2, 4, and 6 µg of SpG were added, basing on the results from the flocculation test (SI). The vis spectra, Z-potential, and dynamic light scattering were acquired at OD ca 1 by diluting the protein-GNP in Milli-Q water. The visible spectra were acquired by using a Varian Cary 1E (Palo Alto, CA, USA) spectrophotometer (wavelength range 480–580 nm, SBW 0.5 nm, rate 900 nm/min) and were reported in the SI (Figure S3). The *Z*-potential and DLS measurements were made by using a DLS Zetasizer Nano ZS90 (Malvern Instruments, Malvern, Worcestershire, UK) instrument (scattering angle 90°, cell temperature = 25 °C, three measurements per sample).

### LFA strip production

The IFNp antigen (1.5 mg/mL) and SpG (0.5 mg/mL) diluted in Milli-Q water were spotted onto nitrocellulose membranes at 1 µL/cm by means of an XYZ3050 platform (Biodot, Irvine, CA, USA) to form the test (TL) and control (CL) lines, respectively. The conjugate pad was pre-adsorbed with the “storage” buffer (see the *Synthesis of the SpA_GNP and SpG_GNP conjugates* paragraph in the SI for details on the pH and the composition) and dried at 60 °C for 1 h. Subsequently, it was dipped into the gold conjugate solution (as diluted in the storage buffer to the reach the appropriate OD) until complete saturation. Then, it was dried at room temperature for 2 h. The membranes were dried at 37 °C for 60 min under vacuum, layered with sample, conjugate, and adsorbent pads, cut into strips (3 mm width) by means of a CM4000 guillotine (Biodot), and inserted into plastic cassettes (Kinbio, Shanghai, China) to fabricate the ready-to-use LFA device. Cassettes were stored in the dark in plastic bags containing silica at room temperature until use.

### Design of experiment for probe selection

The optimal amount of the protein (SpA and SpG) to be adsorbed to 1 mL of GNP (OD1) and the probe amount (measured as the OD of protein-GNP conjugate to be applied to the conjugate pad) were evaluated by means of a full factorial experimental design for each protein separately. The levels of the protein to be adsorbed to GNP were 1, 2, and 4 µg/mL_OD1_ for SpA and 2, 4, and 6 µg/mL_OD1_ for SpG, and the levels of the amount of the probe to be applied to the conjugate pad were OD 2, 3, and 4 for the two probes. The bioligand-to-GNP ratio levels were defined based on the results of the flocculation tests, which results are reported in the SI. In details, levels were selected as the quantity of protein needed to stabilize the GNP; 2 µg/mL and 4 µg/mL are the stabilizing amounts. Therefore, 3 experiments were conducted for each probe. The parameter measured was the intensity of the test line, which was quantified by acquiring strip images with a scanner (OpticSlim 550 scanner, Plustek Technology GmbH, Norderstedt, Germany), and the area of the coloured lines was quantified by means of QuantiScan 3.0 software (Biosoft, Cambridge, UK). For standardization, a goat anti-nucleocapsid protein antiserum was used as representative serum sample that contained anti-N antibodies. The sample was diluted tenfold in the running buffer. In parallel, the absence of non-specific signals was verified for each of the studied conditions and the point discarded in the case of non-specific binding. As the negative control, a pre-COVID serum was used, tenfold diluted in the running buffer. The running buffer used for the checkerboard assay was composed of 115 mM Tris–glycine (pH 8) supplemented with 1% w/v BSA, 2% v/v Tween 20, and 0.02% w/v sodium azide.

### Analysis of human and animal serum samples

Human serum samples, belonging to a panel set from a previous work [13], including 69 rRT-PCR-positive samples and 36 pre-COVID-negative sample, were tested after tenfold dilution in the running buffer. The 36 negative sera were collected before the SARS-CoV-2 outbreak and were made available from the San Luigi Gonzaga Hospital (Orbassano, Torino, Italy). Donors were contacted and provided informed consensus about the use of their specimens. Concerning the positive samples, between 23rd of March and 21st of May 2020, a total of 69 samples from individuals positive to SARS-CoV-2 infection admitted at the San Giovanni Battista, Mauriziano, and Regina Margherita hospitals (Turin, Italy) were included in the study. Positivity was assigned according to the rRT-PCR analysis on a swab sample. After obtaining informed consent, whole blood was collected by venous puncture. Serum was obtained in the same day of collection, immediately heat-inactivated at 56 °C for 30 min and tested using a validated ELISA serological kit (ERADIKIT^TM^ COVID19-IgG). Sera were stored at – 20 °C until analysis. On the day of the analysis, sera were thawed for 30 min at room temperature and gently shaken. Samples were transported and handled in compliance with international standards for biosecurity and biocontainment.

In addition, 4 and 14 samples from suspect dogs and cats, respectively, living with infected and symptomatic people were analyzed. The serum obtainment and treatment was carried as mentioned for human samples. Twenty negative samples collected pre-COVID from pets were also tested.

### Diagnostic performances

Diagnostic sensitivity, specificity, and predictive values were calculated for the two devices based on the visual evaluation of the outcomes from human sera. The concordance between the two serological methods was evaluated by plotting the intensities of the test lines against the ELISA results. Concerning pet sera, only preliminary considerations were extracted, in the absence of a reference diagnosis by PCR.

## Results and discussion

The strips were tested, initially, by applying a goat anti-nucleocapsid protein (GANp) antiserum variably diluted in the running buffer. The proper bioligand-to-GNP ratio for SpA and SpG and the amount of the probe (measured as the optical density of the GNP conjugate, OD) were defined starting from the flocculation test (Figure S1) by using a full factorial design of experiments as described in the Materials and Methods, Design of experiment for probe selection, section. Table 1 shows the localized surface plasmon resonance (LSPR) bands shift for the various SpA_ and SpG_GNP probes prepared in this work, and which differed for the protein-to-GNP ratio. (The visible spectra of the SpA_GNP and SpG_GNP conjugates are reported in Figure S3). The average dimension and *Z*-potential of the probes are also presented from Varian Cary 1E (Palo Alto, CA, USA) and *Z*-potential and dynamic light scattering measurements from DLS Zetasizer Nano ZS90 (Malvern Instruments, Malvern, Worcestershire, UK). The shift of the LSPR peak was similar for all conjugates and slightly larger for SpG_GNP compared to SpA_GNP. In agreement with the LSPR shift, SpG_GNP also showed greater mean diameters and a more negative *Z*-potential, which may indicate a higher stability of these probes, due to a more complete coating of the GNP surface by SpG compared to SpA. Apparently, the same mass of the proteins was needed to reach the saturation of the citrate-capped GNP surface (2 μg of protein per mL of GNP, OD1), even though flocculation tests indicated the need of a larger amount of SpG (4 μg) to shield efficiently GNPs (Figure S1). The recombinant SpG from *Streptococcus* sp. and SpA from *Staphylococcus aureus* used in this study showed molecular weight of ca. 20 kDa and 42 kDA, respectively [28, 29]. Therefore, according to the flocculation test, ca. 0.4 nmol of SpG and 0.048 nmol of SpA were required to stabilize 1 mL of GNPs at optical density of 1. Considering results from DLS and *Z*-potential, the protein-to-GNP molar ratio corresponding to the saturation of GNP surface was higher for SpG (0.1 nmol) compared to SpA (0.048 nmol). The isoelectric point (pI) of SpA is higher (5.1) compared to the one reported for recombinant SpG (4.1–4.2) [28, 29].

**Table 1:** Dimensional analysis and spectroscopic characterization of SpG_ and SpA_GNP probes

Protein amount (μg) per mL of GNP (OD1)		λ_max_ of LSPR (nm)	Average diameter (nm)	*Z*-potential (mV)
*GNP*		525.5 ± 0.5	40 ± 0.5	n.d.^a^
*SpA_GNP*	1	531.0 ± 0.5	51.8 ± 1.1	− 19.2 ± 3.0
	2	531.0 ± 0.5	55.6 ± 0.4	− 23.3 ± 2.3
	4	531.0 ± 0.5	56.6 ± 0.4	− 22.0 ± 1.4
*SpG_GNP*	2	531.5 ± 0.5	60.5 ± 0.8	− 24.3 ± 1.6
	4	531.5 ± 0.5	58.9 ± 0.12	− 25.5 ± 3.4
	6	531.5 ± 0.5	60.0 ± 0.8	− 24.2 ± 2.0

^a^Not detected since the absence of representative overcoating protein in the absence of adsorbed bioreagent

**Fig. 1:**
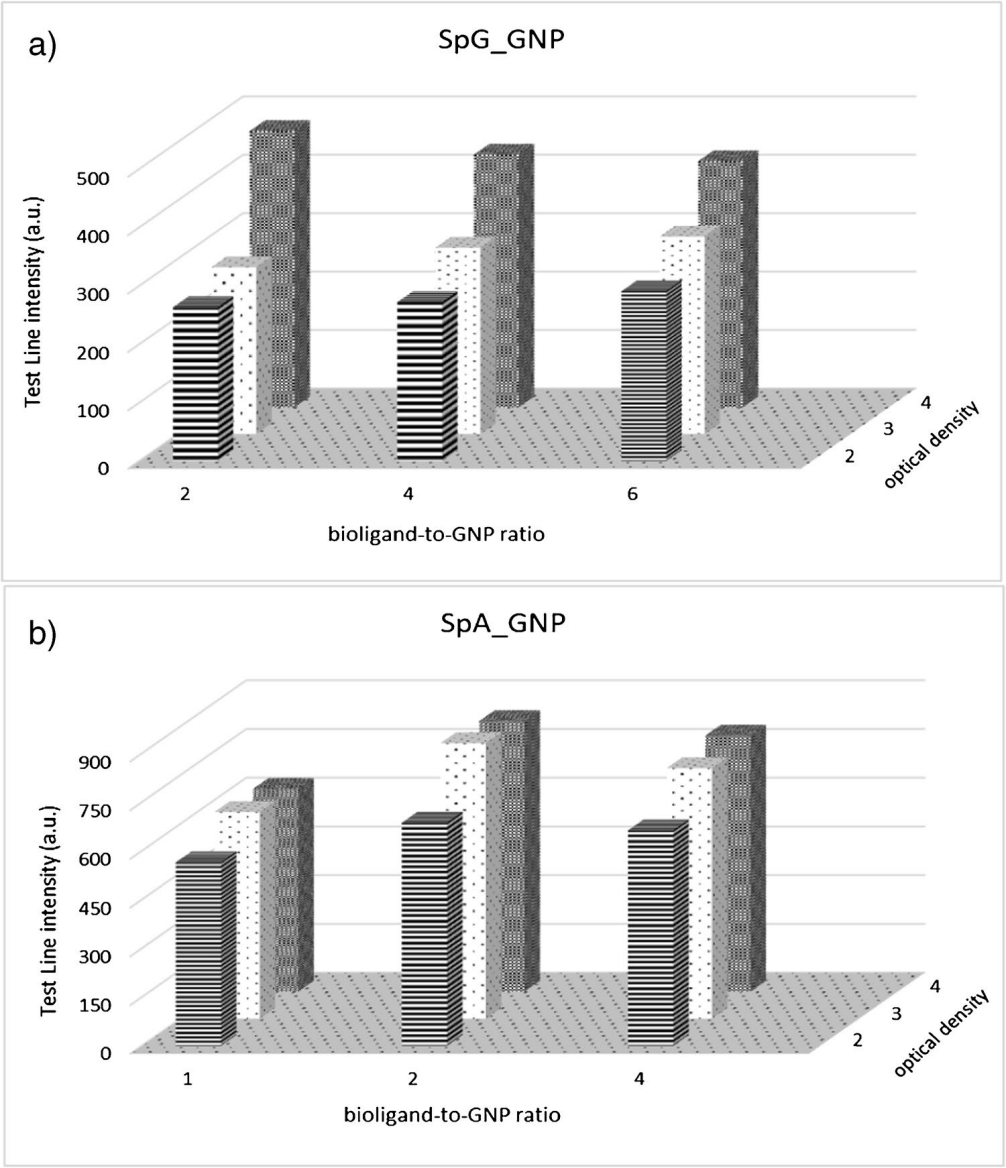
Full factorial design of experiment definition of the proper amount of SpG (**a**) and SpA (**b**) to be adsorbed on the GNPs and the amount (measured as the GNP-conjugate optical density) of the probe

For conjugation to bioligands, GNPs were adjusted to pH 6 so it can be suggested that SpG interacted more efficiently with the GNP surface, due to the larger negative charge at pH 6. Moreover, it seemed that the more SpG adsorbed also guaranteed better stability to the probe. However, as each SpG molecule has two binding sites towards immunoglobulins, the SpG probe owned 0.2 nmol binding capacity. SpA has up to five binding sites, leading to 0.25 nmol of binding capacity for the SpA_GNP probe. Then, the final binding capacity towards immunoglobulins was expected to be almost equivalent. The results of the binding ability of the probes towards immunoglobulins in the LFA platform are reported in Fig. 1, where the colour intensity at the test line was due to the interaction between the probe and human IgG. The complex was captured by the antigen coated to form the test line and was acquired after 15 min from sample application. The binding ability was studied as a function of the bioligand-to-GNP ratio and the optimal probe was defined as a combination of the binding ability and OD of the probe. The highest signals were shown in correspondence of adsorbing 2 µg per mL of GNP for both bioligands and saturating the conjugate pad with the probe amount corresponding to OD4 for SpG (Fig. 1a) and to OD3 for SpA (Fig. 1b). The SpA_GNP showed a distinct saturation effect in the observed ranges of bioligand-to-GNP ratio and OD investigated, as shown by the decrease of the intensity of the test line from OD3 to OD4 for all the bioligand-to-GNP ratios. We supposed that the increasing of the SpA_GNP quantity produced several probes simultaneously bound to the same anti-N immunoglobulin, thus preventing the interaction of the immunoglobulin with the N antigen due to steric hindrance. Another interesting difference between the SpG and the SpA ligands is the different behaviours in the presence of the serum sample. In details, the binding of SpA_GNP to the test line slightly decreased at OD4 while the bioligand-to-GNP ratio increased. We assumed that again the steric hindrance of many immunoglobulins bound to the multivalent SpA impeded the interaction with the N antigen. The SpG has a lower number of binding domains, and the phenomenon does not appear in the explored range of bioligand-to-GNP ratios. Saturation phenomena are generally managed by reducing the amount of the probe or the bioligand-to-GNP ratio. SpA and SpG are relatively small proteins; however, their amount should be carefully optimized as they are multivalent and their interaction with GNPs is very efficient.

Once maximized, the response towards samples fortified with the recombinant antigen, the LFAs were tested on serum samples from subjects with COVID-19 infection, as confirmed by molecular analysis made by means of real-time reverse transcriptase polymerase chain reaction (rRT-PCR).

**Fig. 2:**
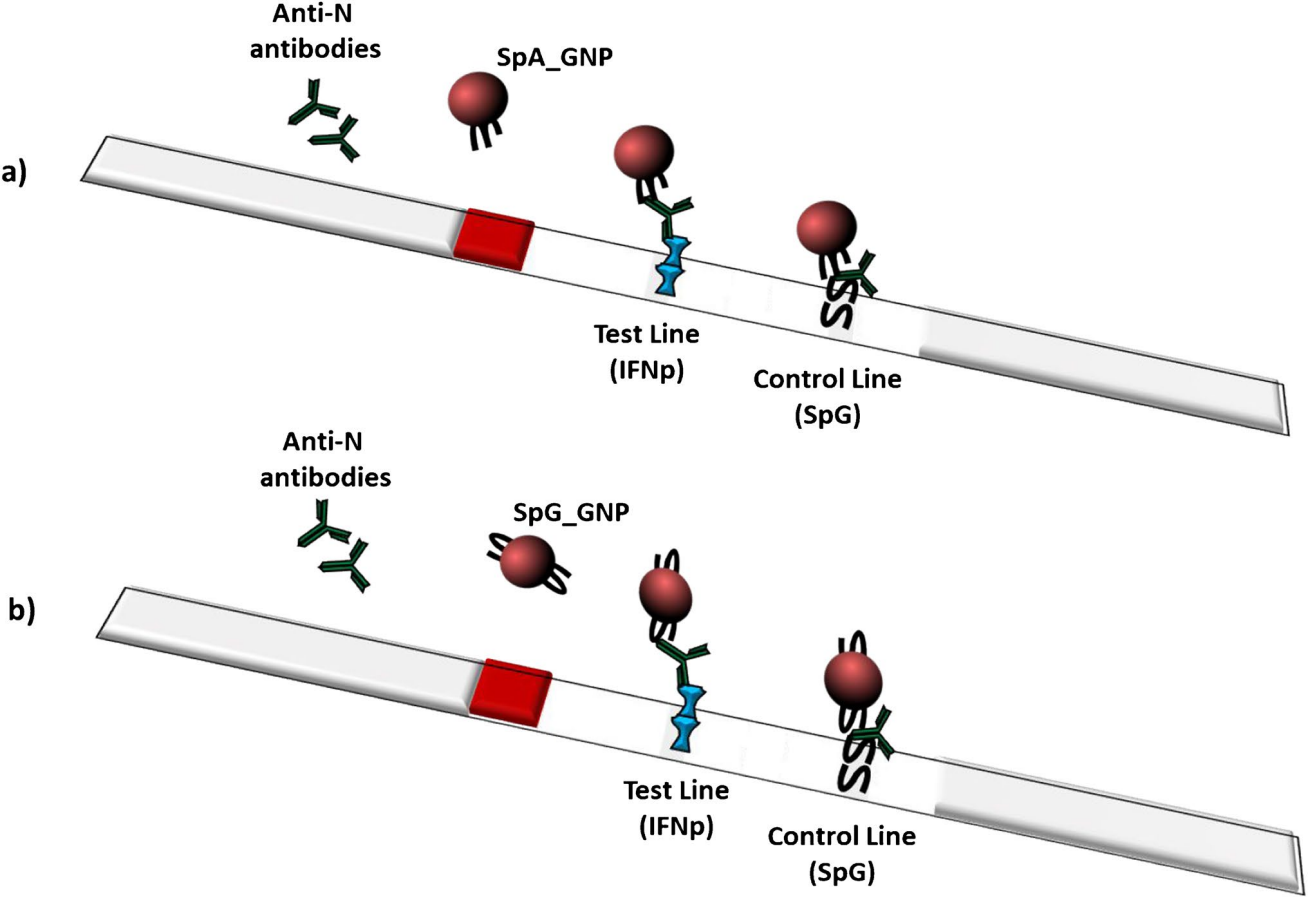
A schematic representation of the A-LFA (**a**) and of the G-LFA (**b**). The sample containing anti-N antibodies encounters the gold conjugate on the conjugate pad and resuspends it. Then, they flow together through the nitrocellulose membrane. On the test line, the anti-N antibodies are captured by the IFNp recombinant antigen. The SpA_GNP (**a**) or the SpG_GNP (**b**) bind to the captured anti-N antibody, and this results in the accumulation of GNPs giving the red colour. On the control line, the SpG captures the antibodies that are similarly detected

The scheme of the two devices and a representation of the assay reactions occurring in the test and control lines are reported in Fig. 2.

The same test and control lines were used in combination with the different labelled bioligands, to avoid other sources of variability between the two devices and to render the comparison more significant. Despite the G-LFA included SpG as the capture and detector ligand in the control line, the line was clearly visible in both the devices, thus indicating that the immunoglobulins present in the serum samples can be captured and revealed by the SpG. We hypothesized SpG, besides its strong affinity to Ig, is also able to cross-react with different portions of the biomolecule, as has been reported [30].

Hereafter, we will refer to the LFA including SpA_GNP as “A-LFA”, to the one including SpG_GNP as “G-LFA”, and to the data obtained in the previous work, which were based on a probe composed by the N antigen linked to GNP, as “N-LFA”. The results from the LFA devices were compared, and their analytical performances were calculated based on the visual output and reported in Table 2. In addition, the signals (colour intensity) as measured at the test lines were obtained by processing images of the strips and were compared to the ones obtained by the N-LFA. The intensity value (arbitrary units, a.u.) below which the human eye cannot discriminate from the background was assessed as 10 a.u. The G-LFA showed lower performances in terms of perceived colour intensity, diagnostic sensitivity (Se = 66.7%), and specificity (Sp = 80.6%) compared to the N-LFA (Se = 81.2%; Sp = 94.4%). The lower sensitivity could be explained considering the extreme selectivity of the bioligand, which limits the reactivity of SpG towards the sole G class of immunoglobulins and the binding capacity in terms of number of binding domains [2]. It should be mentioned that the N-LFA included SpA as the capturing bioligand, to form the test line. On the contrary, the A-LFA showed better performances compared to the N-LFA in terms of diagnostic sensitivity (Se = 89.9%) and a slightly lower specificity (Sp = 91.7%). We reported also almost the same precision (95.4%) of the N-LFA (96.6%) and a higher negative predictive value (82.5%). The plot of the intensity of the colour of the test lines, and the Pearson correlation parameter (0.72) and *R*^2^ (0.52) indicated that the A-LFA is more sensitive than the G-LFA (Figure S4). Unexpectedly, the A-LFA was less correlated to the N-LFA (Pearson correlation 0.58), *R*^2^ 0.33 (Figure S5) despite the two devices fabricated by using the same reagents, which were simply inverted in the role played in the assay (capture or detection). We hypothesized that when applied onto the nitrocellulose, the bioreagents, including SpA, have less degrees of freedom, and this results in a lower overall binding efficiency combined with the short time of contact with the sample. On the contrary, when SpA is used for detection, it encounters the target antigen in solution and the formation of multiple complexes with antigens on different viral particles or repeated epitopes of the same antigen is largely favoured. Another possible interpretation of the higher sensitivity reached using SpA as the detector and the antigen as the capturing agent is connected to the fact that the IFNp antigen does not suffer saturation from non-COVID-specific Igs, which are largely present in the serum. When SpA is used as the capture ligand, the non-specific Igs compete with anti-SARS-CoV-2 Igs for the binding. The G-LFA exploits the same principle but the lower binding capacity on respect to SpA resulted into a lower sensitivity. To confirm the flexibility of the two devices, both were used to test sera from dogs [4] and cats [14]. The results are reported in Table 3. Since no validated assays were available for the diagnosis of COVID-19 in dog and cat sera, the results must not to be considered as true or false positives and negative. In fact, all the animals involved in the study were living into close contact with symptomatic SARS-CoV-2-positive individuals whose infection conditions were confirmed by molecular diagnostic methods [30, 31]. To exclude positive results from non-specific binding, 20 negative samples collected pre-COVID from pets were also tested. Among the 18 animals living with COVID-19-infected subjects, 17 were by the G-LFA and 14 were by the A-LFA rapid test, respectively.

**Table 2:** Diagnostic performances of the G-LFA and A-LFA devices assessed on 69 positive and 36 negative human serum samples (as assigned by rRT-PCR). Data obtained by a N-LFA from the previous work [13] (using N-GNP as the detection antigen and SpA as the capture) was reported to compare the data of the novel devices

	N-LFA	G-LFA	A-LFA
TP	56	46	62
TN	34	29	33
FN	13	23	7
FP	2	7	3
Sensitivity*	81.2 (71.9–90.4)	66.7 (55.5–77.8)	89.9 (82.7–97.0)
Specificity*	94.4 (87.0–100)	80.6 (67.6–93.5)	91.7 (82.6–100)
Precision*	96.6 (91.9–100)	86.8 (77.7–95.9)	95.4 (90.2–100)
NPV*	72.0 (59.6–85.1)	55.8 (42.3–69.2)	82.5 (70.7–94.3)

*P*, positive; *N*, negative; *TP*, true positive; *TN*, true negative; *FN*, false negative; *FP*, false positive; *NPV*, negative predictive value

^*^% (95% CI)

**Table 3:** Results on testing 18 suspected animals living with owner rRT-PCR positive to SARS-CoV-2

Number	Species	Exposure	G-LFA	A-LFA
		(days)	Test line (a.u.)^a^	
1	Cat	27	70	273
2	Dog	32	448	294
3	Cat	44	175	< 10
4	Cat	44	364	147
5	Dog	12	630	175
6	Dog	12	294	< 10
7	Cat	44	217	147
8	Cat	0	343	364
9	Cat	16	770	609
10	Cat	16	259	< 10
11	Cat	30	< 10	< 10
12	Dog	0	462	378
13	Cat	54	210	70
14	Cat	0	203	56
15	Cat	9	133	987
16	Cat	9	224	980
17	Cat	9	168	861
18	Cat	59	56	182

^a^Assessed as positive for higher than 10 a.u

These results are in agreement with previous studies reporting on the possible transmission of COVID-19 to companion animals [24, 32, 33]. The stability of LFA devices was verified by checking the intensity of the test and control lines after 180 days from the production of the strips using a negative sample and the control (GANp). No significant difference was observed compared to the strips used as prepared.

## Conclusions

The two antibody-LFAs proposed in this work were both able to diagnose the immune response to SARS-CoV-2 in humans and companion animals. Though SpA and SpG are often used as broad-specific bioligands in serological LFAs, important differences have been discovered in their performances. SpA appears to be more sensitive than SpG. Furthermore, the selection of the optimal protein-to-GNP ratio was conducted by a full factorial design of experiment based on the selection of the maximum binding of the probe to antigen rather than on the stabilization of GNPs. As observed in previous works [15, 34, 35], the stabilizing amount of the protein is typically not the optimal amount to be adsorbed to GNP to achieve the best sensitivity of the LFA. The calculated diagnostic performances show not only higher sensitivity and but also higher specificity (91.7% (82.6–100)) achieved by using the SpA as the bioligand. By comparing the effect of switching the roles of SpA and N in the N-LFA and A-LFA systems, we observed better performance when using the broad-specific reagent as the detector (A-LFA) compared to drawing it onto the test line (N-LFA). The A-LFA showed improved sensitivity (+ 11%) with a very low loss in specificity (− 3%). These results confirmed previous conclusions [16] on the relevance of design of the assay configuration. The two LFA devices, including non-specific multi-species bioreagents such as SpA and SpG, showed flexibility as they allowed for detecting anti-SARS-CoV-2 antibodies in cat and dog serum samples. These kind of serological antibody tests can be used to check and monitor the immune response followed by the vaccination. Nevertheless, we cannot make a speculation on this application since we are targeting the antibodies against the N protein that is not the antigen used (or encoded by the mRNA) for the vaccination. The presence of this kind of immune response is limited to the former or currently infected individuals. Nevertheless, considering the point of view of the approach, the optimization of the SpA-GNP gold conjugate can be transferred on a serological test employing the spike protein as the capture bioligand, increasing the impact of this work.

## Supplementary Information

Below is the link to the electronic supplementary material.

Supplementary file1 (DOCX 263 KB)
