# Supplementary material for: Bacterial ligands as flexible and sensitive detectors in rapid tests for antibodies to SARS-CoV-2
Source: Anal Bioanal Chem. 2022 Feb 11;414(18):5473–82. doi: 10.1007/s00216-022-03939-2 (PMC8853073; doi:10.1007/s00216-022-03939-2)
Supplement: Supplementary file 1 — Supplementary file1 (DOCX 263 KB) [file 216_2022_3939_MOESM1_ESM.docx]

**Bacterial ligands as flexible and sensitive detectors in rapid tests for antibodies to SARS CoV-2**

Simone Cavalera,*^a^ Fabio Di Nardo,^a^ Matteo Chiarello,^a^ Thea Serra,^a^ Barbara Colitti,^b^ Cristina Guiotto,^c^ Franca Fagioli,^d^ Celeste Cagnazzo,^d^ Marco Denina,^e^ Annagloria Palazzo,^f^ Fiora Artusio,^g^ Roberto Pisano,^g^ Sergio Rosati,^b^ Claudio Baggiani,^a^ and Laura Anfossi.^a^

**Synthesis of Gold nanoparticles**

To prepare gold nanoparticle of approximately 30 nm, 100 ml of 0.01% (w/v) solution of tetrachloroauric acid (HAuCl_4_) in milliQ water were heated to 100°C under vigorous stirring.

1.2 ml of a freshly prepared and filtered 1% w/v solution of sodium citrate were added quickly to the boiling solution. The colour of the solution turned to black and then to ruby red. The solution was stirred under boiling for further 2-3 minutes, then cooled down to room temperature. The so-prepared GNPs were stored at 4°C.

**Flocculation test**

*Figure S1*: The ratio of stable and aggregated gold nanoparticle is plotted towards the amount of protein (µg) added to 1 ml of citrate-capped GNP, OD1. The stable and aggregated GNP fractions were measured as their OD in correspondence of the LSPR peaks of the single (540 nm) and coupled (620 nm) GNPs upon addition of NaCl as aggregation promoter. Error bars represent the standard deviation of two replicate measurements. We considered as stable the concentration in correspondence of the plateau in the trend of the A_540nm_/A_620_nm ratio.

**Synthesis of the SpA_GNP and SpG_GNP conjugates.**

The synthesis of the gold conjugates was made by passive adsorption of the protein (SpA or SpG) on the surface of the citrate-capped GNPs. The conjugation procedure was carried as follows (Figure S2): GNPs solution was adjusted to pH 6 by adding 50 mM carbonate buffer (pH 9.6) and 10 uL of a 0.2mgmL^-1^ solution of SpA were added to 1mL. The SpA was dissolved in 20 mM phosphate buffer (pH 7.4) supplemented with 130 mM of sodium chloride). The mixture was incubated at 37°C for 30 minutes. Then, 100uL of 1% w/v solution of Bovine Serum Albumin (BSA). was dissolved into 80 mM borate buffer (pH 7.4) were added. The overcoating of GNP with BSA was obtained by maintaining the mixture at 37°C for 10 minutes. GNP-protein conjugates were then washed thrice by centrifugation to remove unlabelled SpA or SpG. After each centrifugation, the GNPs pelletized, and the supernatant was delicately discarded. After the first centrifugation, the pellet was resuspended in a 0.1% w/v solution of BSA diluted in 80 mM borate buffer (pH 7.4); after the second centrifugation, the pellet was resuspended in a borate (pH 7.4) “storage” buffer supplemented with 1% w/v of BSA, 2% w/v of sucrose, 0.25% v/v of Tween20, 0.02% w/v of sodium azide; and the third centrifugation was used to concentrate the conjugate to OD>30-40., The pellet was stored at 4°C .


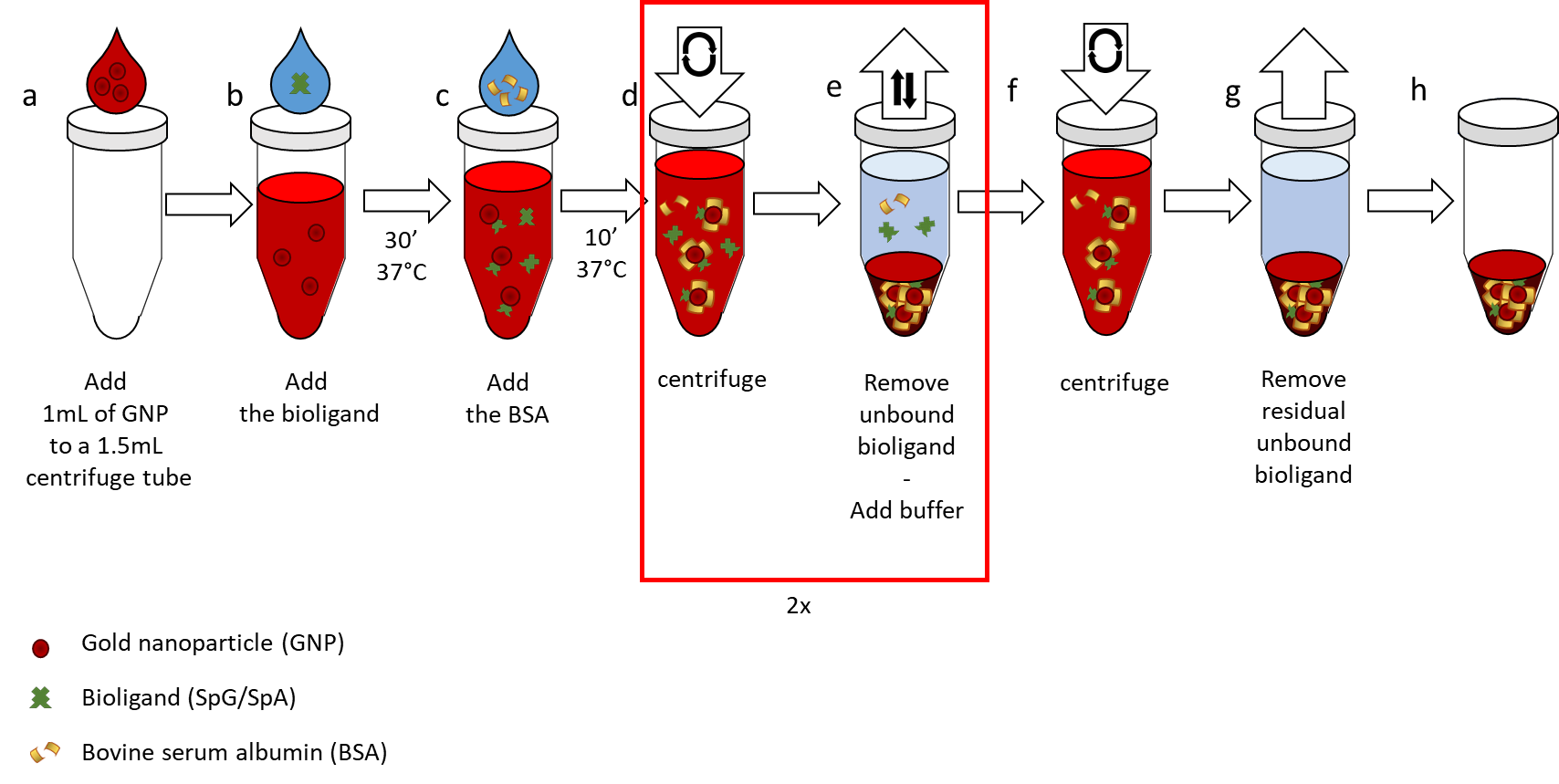


Figure S2: The conjugation protocol for SpA to gold nanoparticles: a) 1 mL of colloidal gold solution at optical density 1; b) SpA or SpG added; c) buffered BSA overcoating of the SpA_GNPs or SpG_GNPs; d) centrifugation; e) elimination of the supernatant containing unadsorbed bioligand and resuspension with buffer; f) centrifugation; g) elimination of the supernatant; h) storage of the SpA_GNPs highly concentrated at 4°C.

**Spectroscopic characterization of SpA-GNPs, and SpG-GNPs conjugates**


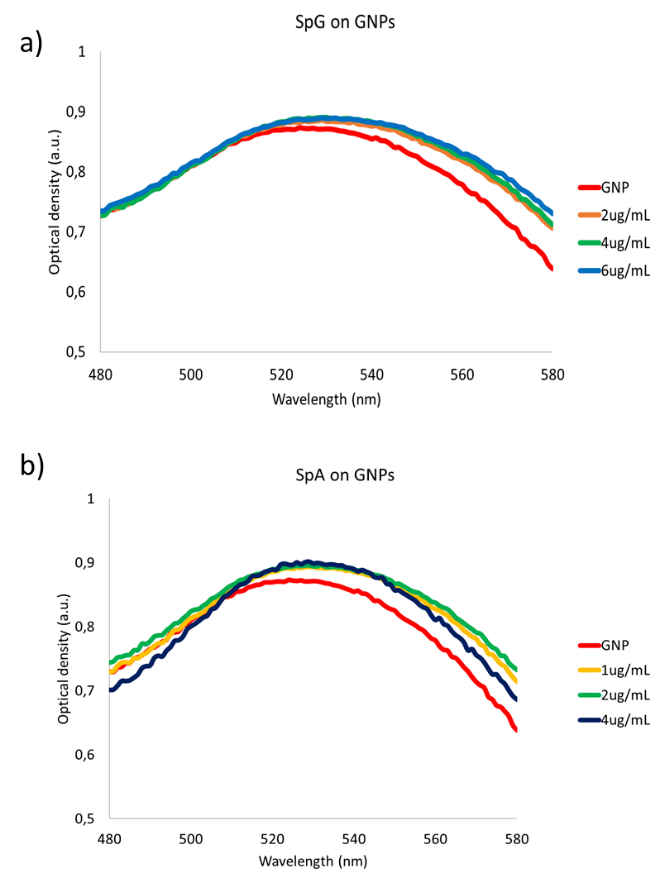


*Figure S3*: The Visible spectra on the SpA_GNP (a) and SpG (b) conjugates at optical density ca1 in milliQ water. In the different conjugates we added to 1mL of GNPs to optical density of 1 a) 0ug (red), 2ug (orange), 4ug (green), 6ug (blue) of SpG and b) 0ug (red), 1ug (yellow), 2ug (green), 4ug (blue) of SpA. The spectra were acquired by using a Varian Cary 1E (Palo Alto, CA, USA) spectrophotometer (wavelength range 480-580nm, SBW 0.5nm, rate 900nm/min).

**Correlation between A-LFA, G-LFA, and N-LFA**

*Figure S4*: correlation between G-LFA and A-LFA on serum samples from a panel set including 69 rRT-PCR+ samples and 36 pre-covid negative sample were tested as 10-fold dilution in the running buffer.

*Figure S5:* Correlation between A-LFA and N-LFA on serum samples from a panel set including 69 rRT-PCR+ samples and 36 pre-covid negative sample were tested as 10-fold dilution in the running buffer.
